# Double Life of Methanol: Experimental Studies and Nonequilibrium Molecular-Dynamics Simulation of Methanol Effects on Methane-Hydrate Nucleation

**DOI:** 10.1021/acs.jpcc.2c00329

**Published:** 2022-03-24

**Authors:** Marco Lauricella, Mohammad Reza Ghaani, Prithwish K. Nandi, Simone Meloni, Bjorn Kvamme, Niall J. English

**Affiliations:** † School of Physics, University College Dublin, Belfield, Dublin 4 D04 V1W8, Ireland; ‡ Istituto per le Applicazioni del Calcolo, Consiglio Nazionale delle Ricerche, 00185 Rome, Italy; § School of Chemical and Bioprocess Engineering, University College Dublin, Belfield, Dublin 4, Ireland; ∥ Dipartimento di Scienze Chimiche, Farmaceutiche e Agrarie (DOCPAS), University of Ferrara, 44121 Ferrara, Italy; ⊥ Hyzen Energy, Laguna Hills, California 92656, United States

## Abstract

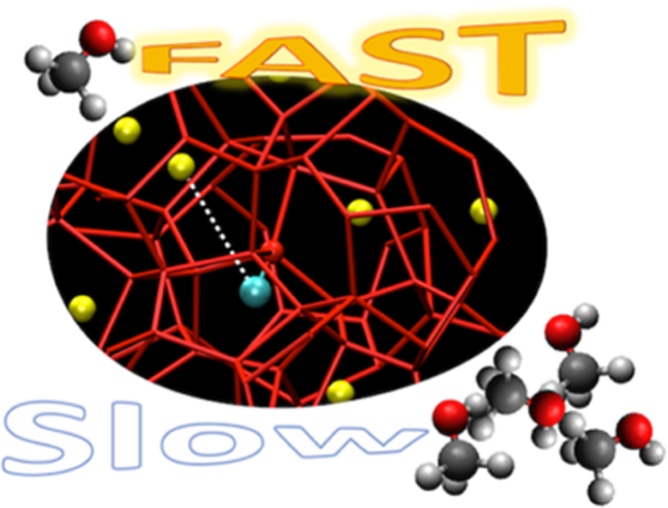

We have investigated systematically and statistically methanol-concentration effects on methane-hydrate nucleation using both experiment and restrained molecular-dynamics simulation, employing simple observables to achieve an initially homogeneous methane-supersaturated solution particularly favorable for nucleation realization in reasonable simulation times. We observe the pronounced “bifurcated” character of the nucleation rate upon methanol concentration in both experiments and simulation, with promotion at low concentrations and switching to industrially familiar inhibition at higher concentrations. Higher methanol concentrations suppress hydrate growth by in-lattice methanol incorporation, resulting in the formation of “defects”, increasing the energy of the nucleus. At low concentrations, on the contrary, the detrimental effect of defects is more than compensated for by the beneficial contribution of CH_3_ in easing methane incorporation in the cages or replacing it altogether.

## Introduction

Clathrate hydrates are nonstoichiometric crystalline inclusion compounds wherein a water host lattice encages small guest atoms or molecules in cavities; hydrogen-bond rigidity confers stability thereto.^1,2^ There are two more common hydrate structures (s)I and II, differing in the type of cavities contained in the unit cell: the sI hydrate features two 5^12^ pentagonal dodecahedral cavities and six slightly larger tetrakaidecahedral 5^12^6^2^ cages^1,2^ and sII features 16 5^12^ cavities and eight medium-sized hexadecahedral 5^12^6^4^ cages.

sI Methane hydrate is the most widespread clathrate type existing in nature in the permafrost and continental-shelf regions and constitutes a possible significant energy resource.^3,4^ Hydrates also constitute a risk for the oil-and-gas industry, as they form under pipeline-operating conditions, with plugging. This makes understanding their formation mechanism important to develop antiplugging strategies. Several hydrate-nucleation mechanisms have been proposed, and molecular simulation and experiments go hand in hand in elucidation thereof:^5−10^ the Labile-cluster hypothesis (LCH)^7^ has proven to be less probable, although the variant local-structuring hypothesis (LHS),^10^ “blob” hypothesis (BH),^11^ and cage-adsorption hypothesis (CAH) are more promising.^8,9,12−16^

Hydrate-nucleation occurs on the microsecond time scale,^5−10,13,17−30^ suggesting that special simulation techniques are necessary to study the process in detail. Lauricella et al. have examined free-energy landscapes for hydrate nucleation from metadynamics^24,31^ and nonequilibrium MD.^23^ Małolepsza et al. applied a generalized replica exchange algorithm for hydrate nucleation.^32^ Bi et al. determined the methane-hydrate nucleation pathway and the free-energy profile by the forward-flux sampling method.^33,34^ Recently, Arjun et al. performed transition path sampling simulations, revealing two possible nucleation channels, indicating that a low-temperature two-step nucleation mechanism, consistent, e.g., with the BH, is replaced by a direct one-step crystallization process at higher temperatures.^35^

The effect of promoters or inhibitors on the underlying details of hydrate nucleation remains unclear. Peters and co-workers have developed frameworks on how additives affect nucleation pathways in general (not specifically in hydrates),^36−38^ noting that substantial accelerations are possible for relatively low dosages. Concerning methanol, Kvamme has argued that the most efficient inhibitory effect arises when alcohol (e.g., methanol) acts as a solvent toward water.^39^ The possibility of low-dosage enhancement of hydrate nucleation by methanol is less explored^36−38^ although it represents an intriguing possibility^39,40^ confirmed by Abay et al.^41^ and Amtawong et al.^42^ FTIR experiments indicate substantial hydrogen bonding of the methanol molecule with “cage walls”,^43^ constraining the convenient methanol orientation vis-à-vis incipient hydrate cages. These intracage interactions outstrip typical van der Waals forces and may well contribute to apparent hydrate-promoting effects.^40^ Simulations were performed to investigate structural characteristics and hydrogen bonding of methanol at the solution–ice interface^44^ and for clathrate hydrates incorporating the additive molecules.^45,46^ Finally, very recently, Su et al.^47^ found that methanol both enhances and suppresses nucleation depending on the temperature of the system. At higher temperatures, above 250 K, methanol competes with water to interact with methane prior to the formation of clathrate nuclei, thus disrupting the formation of water clathrates. Below this temperature threshold, methanol encourages water to occupy the space between methane molecules, favoring clathrate formation. Anticipating our results, here we show that the effect of methanol is more complex and highly depends on its concentration, with its ability to attract methane or replace it altogether in the occupation of the clathrate cavities promoting nucleation when the concentration of the additive in the solution is low.

We remark that while Su et al.^47^ focus on the effect of temperature on the formation of methane clathrate hydrate from a solution at a prescribed concentration of methanol, here we focus on the complementary problem of the effect of concentration at a fixed temperature.

It is very important here to distinguish between methanol effects on heterogeneous hydrate nucleation in the interfacial region in contact with a separate methane phase and nominally homogeneous hydrate nucleation inside water.^48^ The latter is very complicated due to the complex water/methanol structure and how these structures are affected by the presence of methane, which increases methane’s local solubility at the water interface, promoting homogeneous-like hydrate nucleation in the thin layer of water close to the interface with CH_4_.^49^

Typical pipeline methanol levels for flow assurance are ∼40 wt %, and it was found that systems with insufficient methanol (<∼5 wt %) experienced worse plugging than uninhibited systems.^50−52^ This hints at the “double life” of methanol, promoting and inhibiting hydrate nucleation at low and high concentrations, although the mechanistic origins of such a bifurcated character are manifestly unclear.

To address the open methanol-effect questions presented above, the present study applies both nonequilibrium simulations and careful experimental measurements to determine more conclusively methanol-additive effects on methane-hydrate nucleation across a wide concentration range and to ascertain the microscopic mechanisms of the process.

## Methods

### Experimental Setup

**Figure 1:**
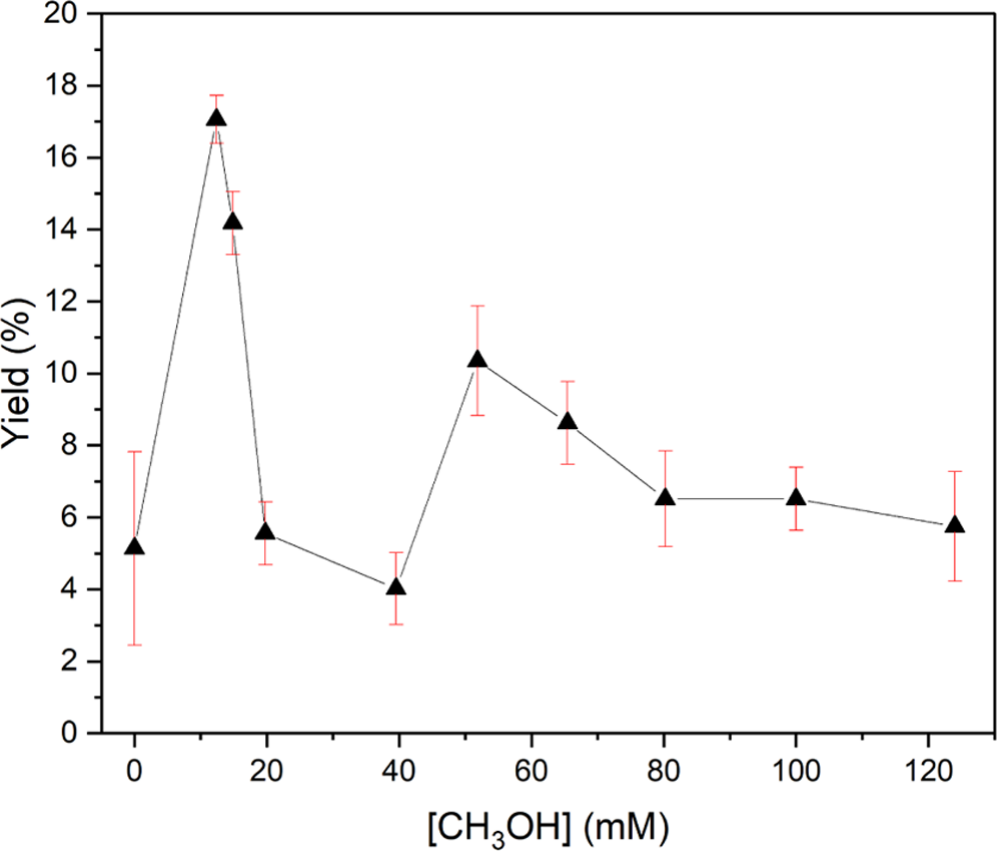
Twenty-four hour enclathration yield (%) as a function of methanol concentration (mol/L). Error bars are also reported, representing 1 standard deviation of the experimental measure. Here, we use molar concentration units for methanol, which are more convenient in the experimental setup, as opposed to molar fraction units used in the rest of the manuscript. We remark that 12 mM, the lowest methanol concentration used in the experiments, corresponds to a mole fraction (χ_CH_3_OH_) of 0.22 × 10^–3^, while 124 mM, the highest concentration, corresponds to χ_CH_3_OH_ = 2.24 × 10^–3^.

We performed tailored experiments to compare against simulation data discussed in the following. Deionized water and varying concentrations of methanol (12–124 mM, or χ_CH_3_OH_ = 0.22–2.24 × 10^–3^) were placed in a temperature-controlled methane-hydrate-formation pressure vessel at 2.9 °C, featuring chemically and heat-treated marine sand, and allowed to form methane hydrate exposed to ∼120 bar methane gas, establishing the inferred 24 h hydrate yield (cf. Figure 1); further details are in the Supporting Information.

### Computational Setup

Simulations were performed following the so-called dynamical approach to nonequilibrium MD (D-NEMD),^53−55^ which uses restrained molecular dynamics (ReMD)^56−58^ for sampling of the initial condition^55^ (see the Supporting Information and ref (23) for further details). We follow nonequilibrium reactive trajectories realizing clathrate-crystallite formation starting from methane–water homogeneous solutions at different supersaturations and at relatively low methanol concentrations. From these trajectories, we compute how nucleation rates depend on the interplay between methane and methanol concentrations. The advantage of this approach with respect to previous works exploiting rare event techniques based on collective variables^24^ is that one does not have to introduce *pulling* observables to drive nucleation, which are difficult to choose for such a complex process,^35^ and in the unfortunate case of a wrong pick, it might alter the mechanism and rate.

For water, we use the “mW” model,^59^ where molecules are represented by point particles interacting through a suitably tuned Stillinger–Weber force field.^60^ mW is impressive for thermodynamic properties but has artificially fast kinetics; however, bearing in mind its low computational cost, mW constitutes an excellent approach for qualitative hydrate-nucleation insights. Methane–methane and water–methane interactions are modeled by two-body terms.^61^ Consistent with the other species, methanol is represented as a two-point particle, with united-atom methane constrained by SHAKE^62^ to a mW water, approximating CH_3_-OH crudely. Surprisingly, this simplistic and computationally efficient model quantitatively reproduces the all-atom characteristics of liquid water/methanol solutions and methane clathrate hydrates incorporating CH_3_OH in the crystal structure (see the Supporting Information). Considering that a single nucleation event with all-atom potential requires ∼1 ms of simulations,^25^ the force model used in this work allows for a good compromise between the feasibility of a rigorous statistical analysis of the nucleation process by D-NEMD and the accuracy of the computational atomistic model.

The computational sample consisted of a two-phase orthorhombic simulation box, with methane reservoirs providing the gas to the solution in the box’s central part. Four values of the solvated-methane mole fraction, χ_CH_4__, were considered: 0.038, 0.044, 0.052, and 0.058, labeled A, B, C, and D, respectively. We added three different methanol quantities to A–D, χ_CH_3_OH_ of 0, 4, 8, and 16 × 10^–3^, leading to 16 systems, with 40–80 independent trajectories each (see the Methods section and the Supporting Information for additional details).

## Results and Discussion

In broad terms, Figure 1 shows a significant nucleation enhancement vis-à-vis the zero-methanol case at low concentrations. This is consistent, though complementary, with previous experiments on powdered frozen water/methanol solid solutions exposed to methane gas at 30–125 bar and 253 K.^44^ The nucleation enhancement reduces at higher concentrations, lending further direct evidence of methanol’s concentration-dependent promotion-inhibition dichotomy.^50−52^ χ_CH_3_OH_ < 0.35 × 10^–3^ serves to promote hydrate formation, which declines as the methanol concentration increases. There is a reverse trend in the 40–50 mM range (χ_CH_3_OH_ < 0.7–0.9 × 10^–3^), where additional methanol increases the hydrate yield; however, this trend changes to a more modest promotion vis-à-vis the no-methanol case above χ_CH_3_OH_ ∼ 0.9 × 10^–3^, which is consistent with previous works. Indeed, three distinct régimes of pressure drop were observed during hydrate formation (cf. Figure S6), analogous to literature results,^44,45,50−52^ interpreted by the “shrinking-core” model.^52^ In the present work, each stage’s duration is plotted in Figure S6 for different methanol concentrations: the first lasts roughly 45 min for all concentration values. Stage II is considered crucial, while there is a direct relation between hydrate-formation yield and duration; stage III correlates inversely with yield.

In the simulations, it was observed that hydrate nucleation occurred very quickly with χ_CH_4__ = 0.044–0.058, with negligible induction time: at high concentrations, the system is essentially already a supercritical blob readily evolving toward the corresponding clathrate. The methanol-concentration effects on hydrate-formation kinetics for these cases—whether it inhibits or enhances nucleation—are irrelevant. Thus, we focus here primarily on χ_CH_4__ = 0.038, where we found meaningful and important methanol effects.

**Figure 2:**
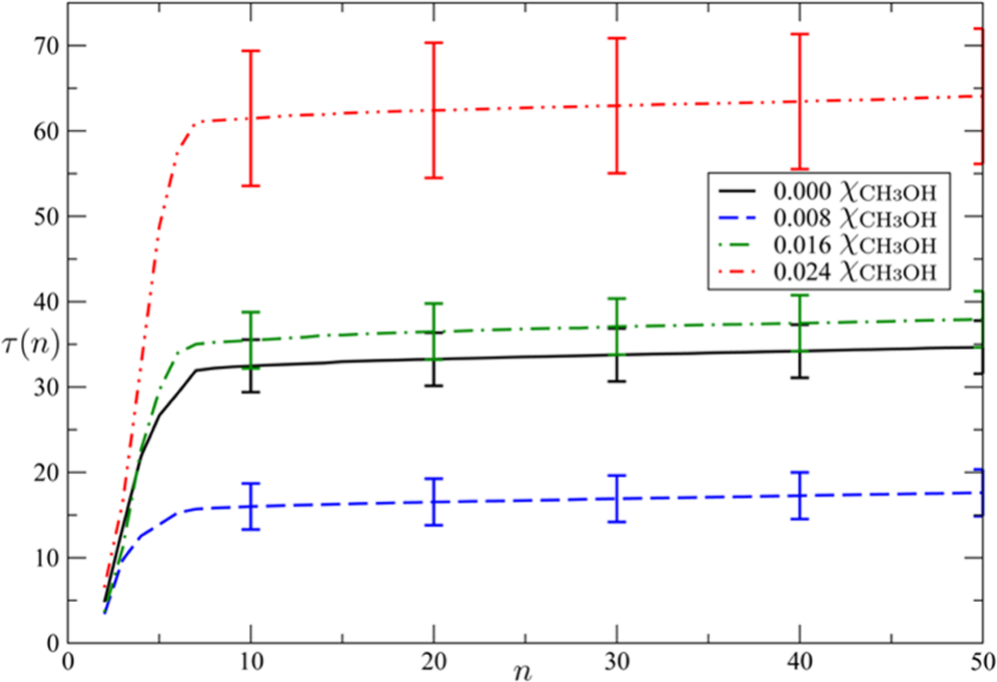
First mean passage time τ vs number of clathrate cages from MD fitted to eq 1 for several values of methanol mole fraction. One notices that for χ_CH_3_OH_ = 0.008 the clathrate-nucleation time is shorter than in the case without any additive, i.e., χ_CH_3_OH_ = 0.008 promotes nucleation. For χ_CH_3_OH_ = 0.024, on the contrary, nucleation is slower, i.e., at this concentration, methanol is an inhibitor. χ_CH_3_OH_ = 0.016, a concentration at which the nucleation time is the same for pure water within the error bars, is the crossover concentration between enhancement and inhibition concentrations.

To analyze simulations, we follow the total number of complete hydrate cages *n*_cages_(23,24,31) along D-NEMD trajectories. Clathrate-nucleation kinetics was analyzed via first-passage time (FPT): *t*(*n*) = inf{*t* > *t*_0_|*n*_cages_(Γ(*t*)) > *n*}, i.e., the first time the nucleus consists of more than *n* cages. Nucleation rates can be evaluated using the mean first-passage time τ(*n*) (MFPT),^23^ the average value of *t*(*n*) over independent realizations of nucleation. If the nucleation barrier is sufficiently high, τ(*n*) is given accurately by^23^
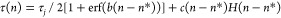
1where the second term in the r.h.s. is a heuristic term taking into account CH_4_ diffusion-limited clathrate formation. Here, τ_*j*_ is the nucleation time, *b* is related to the Zeldovich factor (*Z* = *b*/√π), n* is the critical size, *H*(•) is the Heavyside step function, and *c* = 1/*ν*_g_ is the inverse of the formation rate (*v*_g_ = ∂*n*/∂τ). We report, in Figure 2, τ vs *n* for simulations at χ_CH_4__ = 0.038 for χ_CH_3_OH_ = 0, 0.008, and 0.024; the intermediate concentration (χ_CH_3_OH_ = 0.016) shows an overlap with the pure-methane case (see Figure S8 for the other cases). Therefore, consistent with the experiment in Figure 1 and refs (48−51), Figure 2 shows, prima facie, that χ_CH_3_OH_ of 0.024 inhibits hydrate-formation kinetics, while 0.008 promotes it (with nonoverlapping error bars, rejecting *H*_0_ in two-tailed Student’s *t*-tests to over 95% confidence level).

Although D-NEMD and experiments have been conducted at different temperatures and pressures, with more aggressive temperature and pressure driving forces needed in MD for ostensibly-homogeneous nucleation, as opposed to primarily heterogeneous nucleation in experiments, the positive correlation between simulation and experiment methanol’s hydrate-formation “double life” is rather striking. The different conditions, heterogeneous vs homogeneous, and pressure and temperature certainly call for a future confirmation of this positive correlation. Nevertheless, we believe that present results stimulate research and discussion on this theme.

**Figure 3:**
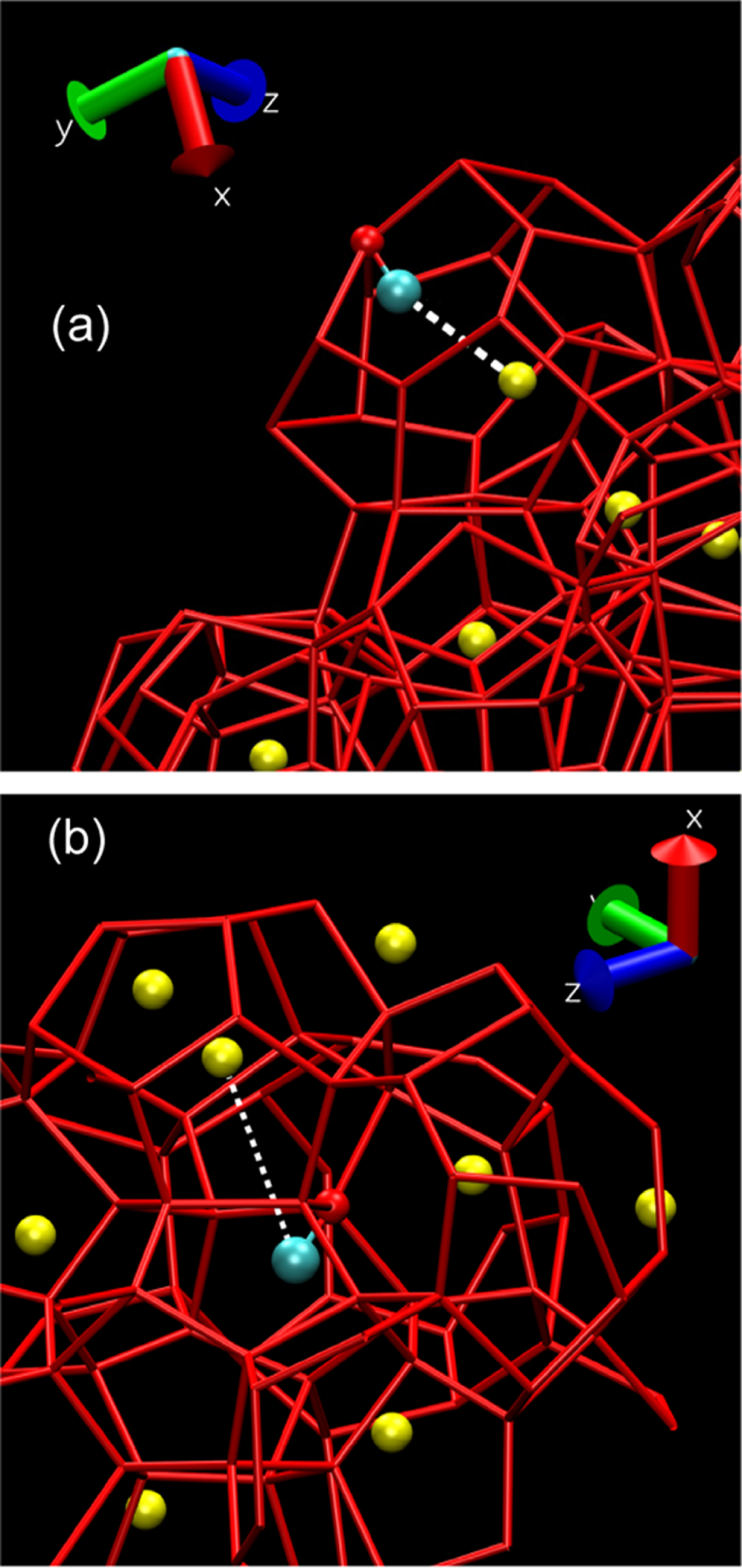
Selected MD snapshots showing the (a) presence and (b) absence of methane in cavities featuring “inward-pointing” methanol-methyl groups. Red sticks represent hydrogen bonds connecting (mW) water molecules at the corners of the clathrate hydrate framework, yellow spheres represent methane molecules, and blue spheres represent the methyl group of methanol molecules. In panel a, one notices that there is a methane molecule inside the clathrate cage hosting the methyl group of the methanol incorporated in the clathrate framework, as highlighted by the short distance between the two groups (white dashed line). Panel (b) shows a case in which, on the contrary, the cage hosting the methyl group of methanol does not contain any methane molecule, and the distance with the closest CH_4_ is much larger than in the previous case.

**Figure 4:**
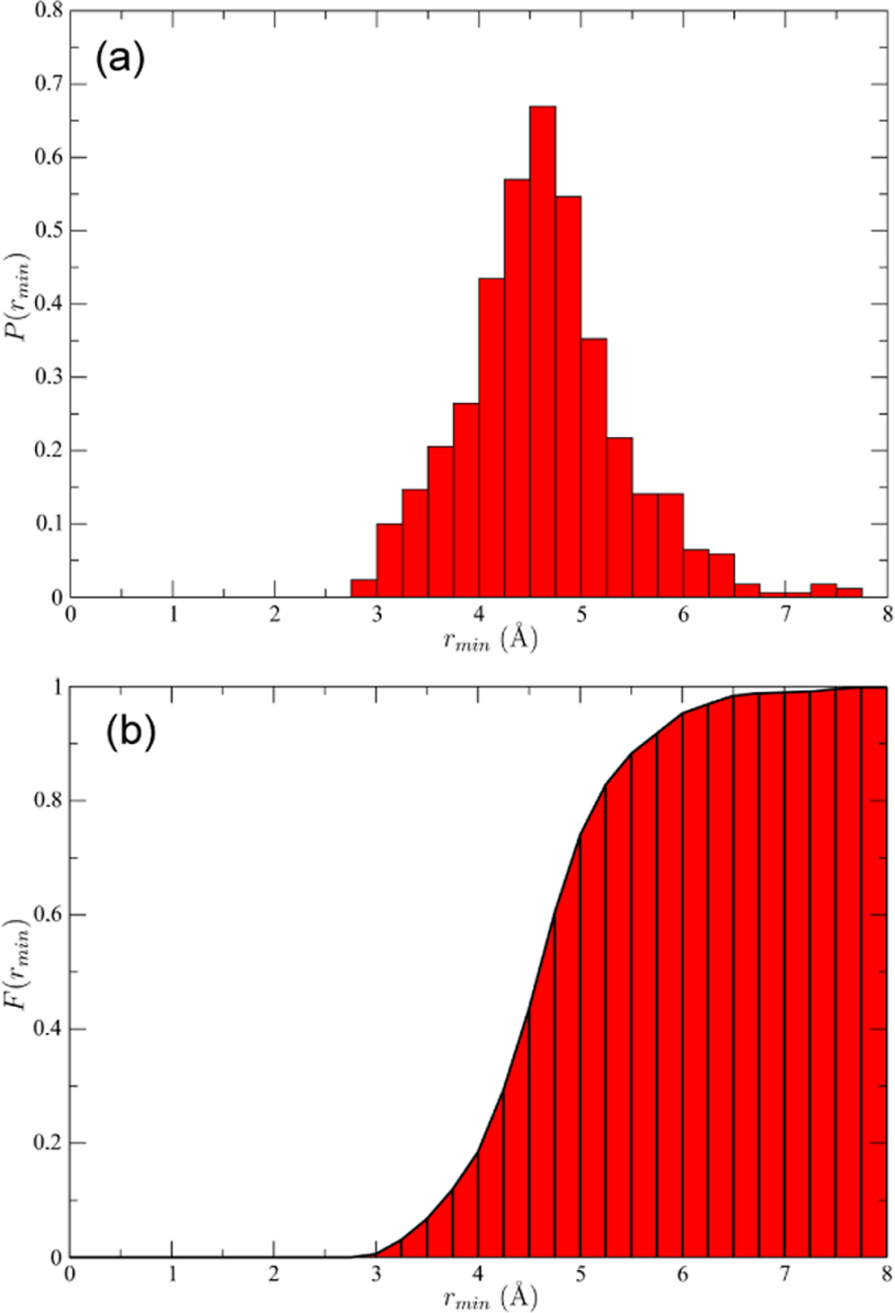
(a) Normalized probability-density distribution, together with (b) cumulative probability distribution (i.e., integral of the first part), of the minimal distance of methanol-carbon to methane molecules, i.e., from each methanol-carbon atom to its nearest methane neighbor.

Probing mechanistic hints for methanol’s “double life,” the key observation is that, under certain conditions, methanol molecules can become part of the hydrate lattice itself, forming hydrogen bonds with their OH groups, which is consistent with previous experimental observations.^45^Figure 3 depicts representative examples of methyl groups pointing inward into cages, featuring both the presence (Figure 3a) and absence (Figure 3b) of methane molecules therein. Naturally, there is some distortion of rings/cavities, owing to the reduced hydrogen-bonding coordination in methanol’s OH groups. In Figure 4, methanol–methane nearest-neighbor distributions, with just ∼10% of these pairs within a distance compatible with occupation of a cage by both methane and the hydrophobic tail of methanol, show that for about 90% of the cages the latter replaces the former in stabilizing clathrate cavities.

Thanks to its amphiphilic nature, methanol either helps embed methane in a local environment consistent with 5^12^ and 5^12^6^2^ clathrate hydrate cages, which facilitates clathrate nucleation, or itself plays the double role of the element of the hydrogen-bond network of the crystal nucleus and hydrophobic filling of the cavities, also in this case facilitating nucleation. However, methanol incorporation in the clathrate structure, with its lower coordination, introduces defects in the hydrogen-bonding structure of the growing crystal, thus increasing the energy of the nucleus of the prescribed number of cages. Indeed, at low concentrations, with methanol’s liquid-phase chemical potential lower, it can more easily (or quickly) leave the growing clathrate nucleus, fostering crystallite growth. The fraction of methanol incorporated in the nucleus structure grows with methanol concentration in the solution (Figure S2) and so does its hydrogen-bond network defect. This likely results in an increase in energy of the crystallite and hence in an increase of the nucleation barrier. Indeed, MD analysis shows that the critical-nucleus size per se did not change markedly with methanol concentration, while the nucleation rate does (cf. Figure 2)—consistent with the nucleation barrier, itself connected intimately to τ_*j*_ (vide supra), shrinking—supporting the idea of critical-nucleus stabilization, in keeping with the overall shrinking-core model.^63^ Given the small size of the clathrate critical nucleus, ∼2–4 cages (Figure 2), the stabilization effect of a low concentration of methanol filling or attracting methane to fill a few cages can significantly enhance nucleation.

This broader “double life” interpretation cannot be explained solely by methanol’s in-lattice incorporation; we admit that Figures 3 and 4 do show rather striking events deserving further future atomistic-force field investigation.

## Conclusions

In closing, we present mechanistic and statistical insights into methanol’s “double life” vis-à-vis hydrate-nucleation effects, noting promotion at low concentrations and switching to industrially familiar inhibition at higher concentrations and providing a mechanistic origin of the phenomenon. Further molecular-simulation studies with fully atomistic force fields will be performed together with spectroscopic work to confirm the relevance of in-lattice methanol incorporation for clathrate-nucleation suppression/enhancement.

## Supporting Information Available

Theoretical background; validation of the atomistic model; computational details; experimental details; and additional MFPT vs n_cages_ curves (PDF)
