# Supplementary material for: Double Life of Methanol: Experimental Studies and Nonequilibrium Molecular-Dynamics Simulation of Methanol Effects on Methane-Hydrate Nucleation
Source: J Phys Chem C Nanomater Interfaces. 2022 Mar 24;126(13):6075–81. doi: 10.1021/acs.jpcc.2c00329 (PMC8996238; doi:10.1021/acs.jpcc.2c00329)
Supplement: Supplementary file 1 — jp2c00329_si_001.pdf [file jp2c00329_si_001.pdf]

Supporting Information

for

The Double Life of Methanol: Experimental Studies and Non-Equilibrium Molecular-Dynamics Simulation of Methanol Effects on Methane-Hydrate Nucleation

Marco Lauricella[*a,b], Mohammad Reza Ghaani*[*c], Prithwish K. Nandi*[c†], Simone Meloni*[*a,d], Bjorn Kvamme*[e] and Niall J. English*[*c]*

a School of Physics, University College Dublin, Belfield, Dublin 4, Ireland.

b Istituto per le Applicazioni del Calcolo, Consiglio Nazionale delle Ricerche, 00185 Rome, Italy

c School of Chemical and Bioprocess Engineering, University College Dublin, Belfield, Dublin 4, Ireland.

d Dipartimento di Scienze Chimiche, Farmaceutiche e Agrarie (DOCPAS), University of Ferrara, 44121 Ferrara, Italy.

e Hyzen Energy, Laguna Hills, CA, 92656, USA

~~The Double Life of Methanol: Experimental Studies and Non-Equilibrium Molecular-Dynamics Simulation of Methanol Effects on Methane-Hydrate Nucleation~~

~~Marco Lauricella * [a,b], Mohammad Reza Ghaani * [c], Prithwish K. Nandi [c]+, Simone Meloni * [a,d], Bjorn Kvamme [e] and Niall J. English * [c]~~

~~*a) School of Physics, University College Dublin, Belfield, Dublin 4, Ireland.*~~

~~*b) Instituto per le Applicazioni del Calcolo, Consiglio Nazionale delle Ricerche, Rome, Italy*~~

~~*c) School of Chemical and Bioprocess Engineering, University College Dublin, Belfield, Dublin 4, Ireland.*~~

~~*d) Dipartimento di Scienze Chimiche e Farmaceutiche, University of Ferrara, Ferrara, Italy.*~~

~~*e) Dept. of Physics and Technology, University of Bergen, Allegaten 55, 5007, Bergen, Norway.*~~

Dynamical nonequilibrium molecular dynamics

Dynamical nonequilibrium molecular dynamics, D-NEMD,[1,2] allows to compute the expectation value of observables at time t after the system is left to evolve starting from an initial “macroscopic” condition. Macroscopic condition, here, must be understood as the statistical mechanics ensemble associated to some value of a suitable set of observables, which can be the same or different from the ones one follows along the time evolution of the system. The system can be prepared in an initial macroscopic condition by, for example, restrained molecular dynamics, ReMD,[3–6] which is described in the next section.

More in detail, let us denote $O(\Gamma)$ a generic observable which is a function of the phase space Γ. For the time-dependent case, the observable $\bar{O}(t)$ is an ensemble average in phase space of $O(\Gamma)$

$$\bar{O}(t)=\int_{\Gamma} O(\Gamma) f(\Gamma,t)\,\mathrm{d}\Gamma \tag{1}$$

where $f(\Gamma,t)$ is the time-dependent probability density function, PDF, satisfying the Liouville equation $\partial f(\Gamma,t)/\partial t = -\{f(\Gamma,t),H(\Gamma,t)\} = -iL(t)f(\Gamma,t)$, with $\{\cdot,\ \cdot\}$ denoting the Poisson bracket and L(t) the Liouville operator. Since numerical evaluation of $f\ (\Gamma,\ t)$ cannot be computed explicitly, apart from simple cases, we apply the Onsager-Kubo relation

$$\bar{O}(t)=\int_{\Gamma} O(\Gamma) S^*(t) f(\Gamma,t_0)\,\mathrm{d}\Gamma=\int_{\Gamma} S(t) O(\Gamma) f(\Gamma,t_0)\,\mathrm{d}\Gamma \tag{2}$$

~~Corresponding authors: * lauricella.marco@gmail.com, mlnsmn@unife.it, mohammad.ghaani@ucd.ie, niall.english@ucd.ie~~
~~+ Current address: Irish Centre for High-End Computing, Trinity Enterprise Tower, Pearse St, Dublin 2, Ireland~~

where $S\,(t)$ is the time-evolution operator, $S^*(t)$ is its adjoint, and $f\,(\Gamma,\, t_0)$ is the PDF at the initial time t_0. In so doing, we can compute the time-dependent average $\overline{O}(t)$ as the ensemble average over the initial PDF $f\,(\Gamma,\, t_0)$ of the observable $O(\Gamma(t))$ at the point $\Gamma\,(t)$, corresponding to the evolution in time of the initial phase-space point $\Gamma\,(t_0)$. In practice, in the D-NEMD approach, we sample initial positions and velocities from the initial PDF $f\,(\Gamma,\, t_0)$ by ReMD, typically between 40 and 80 per computational sample, and evolve them in time along relaxation trajectories obtained by MD governed by the physical Hamiltonian $H\,(\Gamma)$. Along these trajectories, we compute the observable $O\,(\Gamma(t),\, t)$ and average it among values obtained on the various trajectories at the same time *t,* thus obtaining $\overline{O}(t)$ for the given sample.

Restrained molecular-dynamics

Nonequilibrium molecular dynamics requires to sample the time dependent probability density function, PDF, starting from an initial distribution at time t=0 associated to some prescribed values of a set of observables. This initial distribution can be sampled through restrained molecular dynamics, ReMD.[3–6] let us consider a classical system of N particles at constant temperature T and let us denote the phase space of the system by Γ. We want to sample the constant number of particles, volume and temperature (NVT) distribution with the additional constraint that an observable $O(\Gamma)$ takes the value O^*, $f(\Gamma|O(\Gamma)= O^*)$. Here, for example, the observable O is a suitable estimator of the solvated methane and methanol molar fraction. By definition

$$f\left(\Gamma|O(\Gamma)=O^*\right)=\frac{f(\Gamma)\,\delta\left(O(\Gamma)-O^*\right)}{\int_{\Gamma} f(\Gamma)\,\delta\left(O(\Gamma)-O^*\right)\mathrm{d}\Gamma} \tag{S1}$$

where $f\,(\Gamma)$ is the PDF without extra conditions; here, $\delta(\bullet)$ is the Dirac's delta enforcing the condition $O(\Gamma)= O^*$. As described by Maragliano *et al.*,[3] this distribution can be sampled by a molecular dynamics driven by an extended Hamiltonian $H_k(\Gamma)$, consisting in the sum of the physical Hamiltonian $H_0(\Gamma)$ plus a biasing quadratic potential:

$$H_k(\Gamma)=H_0(\Gamma)+\frac{k}{2}\left(O(\Gamma)-O^*\right)^2 \tag{S2}$$

where k is a tunable parameter. The corresponding canonical PDF is

$$f_k(\Gamma) = \frac{\exp\left[-\beta H_0(\Gamma) - \beta \frac{k}{2}\left(O(\Gamma) - O^*\right)^2\right]}{\int_\Gamma \exp\left[-\beta H_0(\Gamma) - \beta \frac{k}{2}\left(O(\Gamma) - O^*\right)^2\right] \mathrm{d}\Gamma} \quad \text{(S3)}$$

Noting that $\lim_{k\to\infty} \exp\left[-\beta \frac{k}{2}\left(O(\Gamma) - O^*\right)^2\right] \Big/ \sqrt{2\pi/\beta k} = \delta\left(O(\Gamma) - O^*\right)$, so that in the limit of $k \to \infty$, we observe the right-hand side of eqn. S3 going to eqn. S1, and we obtain $f_k(\Gamma) \to f\left(\Gamma | O(\Gamma) = O^*\right)$. Restraining molecular dynamics must not be confused with molecular dynamics using to relax the initial condition; In ReMD, using the terminology of statistical mechanics, time is used for *sampling*, while in the relaxation trajectory time preserves its genuine meaning.

We wish to sample nucleation events at different supersaturated conditions of methane and (relatively low) solvated-methanol concentrations; thus, we need to introduce observables counting number of solvated methane and methanol molecules in water solution, n_{CH4} and n_{CH3OH}, respectively. The estimator $n_{\mathrm{CH4}}(\Gamma)$ is defined as

$$n_{\mathrm{CH}_4,\,a}(\Gamma) = \sum_{i=1}^{n_{\mathrm{met}}} \varphi_a(\Gamma) \quad \text{(S6)}$$

where φ_a is a characteristic function associated to the ath sub cell. In other words, the molecule contributes with 1 to n_{CH4} when it is in the ath sub cell and 0 otherwise. This definition follows that given by Irving and Kirkwood for a microscopic field.[7] In our case, we define a unique sub cell, φ, as a central orthogonal sub volume delimited along the *z*-axis by two perpendicular flat boundaries, located at z_1 and z_2 with $z_1 < z_2$, while length and width are the same of the simulation cell. For this cell, a simple form of the characteristic function is

$$\varphi(z_i) = \Theta(z_i - z_1)\,[1 - \Theta(z_1 - z_2)] \quad \text{(S7)}$$

where z_i is the component of the ith particle along the *z* axis in the ordinary space, and $\Theta(\cdot)$ is the Heaviside step function. It is worth stressing that the Heaviside step function is a discontinuous function of its domain, resulting to impulsive forces on atoms crossing the boundary of the φ cell due to the biasing term. This problem is usually addressed by replacing $\Theta(\cdot)$ by a smooth analytical approximation, here

$$\Theta(x) \approx \frac{1}{\exp(-\lambda x) + 1} \quad \text{(S8)}$$

where λ is a parameter controlling the smoothness of the approximation.[8,9]

At high methane-supersaturation conditions, we note that the solution tends not only to relax towards the final hydrate state, but a significant part of solvated methane is involved in the nucleation of pure methane bubbles. This is mainly due to the low solubility of methane in water. The consequences are essentially twofold: (1) the homogeneity of the solution is not preserved, and (2) the concentration of solvated methane decreases in solution. Hence, we need to introduce a further parameter to avoid the formation of pure methane bubbles in the sub cell φ. Following previous successful works,[10–12] we exploit the difference in the coordination number of the pair CH_4-CH_4 at distance equal to 5.5 Å (in the following text denoted by $CN_{\text{CH4–CH4}}$) between the bulk liquid solution, and the supercritical fluid of pure methane in the bubble.[10,13] With neighboring cutoff distance 5.5 Å, $CN_{\text{CH4–CH4}}$ is close to zero for the bulk solution whilst it is greater in the pure methane bubble. Thus, $CN_{\text{CH4–CH4}}$ can be used to discern/prevent the presence of pure methane bubbles in solution. Consistently with n_{CH4}, we define a suitable estimator of $CN_{\text{CH4–CH4}}$ as

$$CN_{\text{CH4-CH4}}(\Gamma) = \frac{1}{N_c} \sum_{i=1}^{N_{\text{CH}_4}} \sum_{\substack{j=1 \\ j \neq i}}^{N_{\text{CH}_4}} \frac{1}{\exp\left[\lambda\left(r_{ij}(\Gamma) - R_{\text{cut}}\right)\right] + 1} \qquad \text{(S9)}$$

where the argument of the summation is the smooth analytical approximation of $\Theta(\cdot)$ already introduced in eqn. S8. R_{cut} is equal to 5.5 Å, and N_c is a suitable scalar factor. Using the ReMD approach, we restrain the value of $CN_{\text{CH4–CH4}}$ around zero so that the homogeneity of the solution and the concentration of solvated methane are preserved during the time trajectory. It is worth stressing that the extended Hamiltonian introduced in eqn. S2 within the ReMD approach is now composed of three biasing quadratic potential terms, which restrain the time evolution around the target values of n_{CH4}, n_{CH3OH} and $CN_{\text{CH4–CH4}}$, respectively.

To apply D-NEMD, we need to sample a proper stationary condition ensemble to assess the initial PDF $f(\Gamma, t_0)$. In our case, the initial conditional ensemble describes a state consisting of an aqueous supersaturated phase of solvated methane. Thus, we need a suitable observable able to characterise the initial state and distinguish it from the final hydrate state of equilibrium. Cluster-analysis hydrate-recognition methods provide non-analytical functions of phase space, and, consequently, cannot be used in the ReMD approach. Therefore, we use a revised analytical version of the parameter F_4, already proposed by Rodger *et al.*[14] The parameter F_4 is defined as

$$F_4 = \frac{1}{N_c} \sum_{i=1}^{N_{\text{H2O}}} \sum_{j=1}^{N_{\text{H2O}}(i)} \sum_{k=1}^{N_{\text{H2O}}(j)} \sum_{l=1}^{N_{\text{H2O}}(k)} \cos\left(3\xi_{i,j,k,l}\right) \qquad \text{(S10)}$$

where N_c is a scalar factor and $\xi_{i,j,k,l}$ is the dihedral angle, defined as the angle between two planes: the first plane is determined by the two non-collinear vectors $\mathbf{r}_{i,j}$ and $\mathbf{r}_{j,k}$ and the second is determined by the non-collinear vectors $\mathbf{r}_{j,k}$ and $\mathbf{r}_{k,l}$, where $\mathbf{r}_{i,j}$, $\mathbf{r}_{j,k}$ and $\mathbf{r}_{k,l}$ are the vectors connecting four water molecules labelled *i*, *j*, *k*, *l*.[14] $N_{H2O}(i)$ denotes the number of water molecules within a spherical shell centred on the ith water molecule within a radius of 3.5 Å, corresponding to the position of the first minimum in the water-water (O-O) pair correlation function. The $\xi_{i,j,k,l}$ dihedral angles in liquid water take on all possible values, with a 25 % difference between the most (60°, 180°, and 300°) and least (120° and 240°) probable angles.[15] Thus, F_4 in bulk water is zero. In all crystalline structures where water molecules form almost planar 5- and 6- membered rings, F_4 adopts non-zero values, since the most probable angle is 180°.[14] To sample the initial PDF f (Γ, t_0) via D-NEMD, we add another biasing quadratic potential term to the extended Hamiltonian of eqn. S2 to restrain the parameter F_4 around zero, corresponding to the bulk solution. We stress that this term is active only during sampling of the initial PDF f (Γ, t_0).

<u>*Computational Details*</u>

We prepared 16 different computational samples arising from the combination of four concentrations of methane (χ_{CH4}=0.038, 0.044, 0.052, 0.058, denominate samples A, B, C and D) and four concentrations of methanol (χ_{CH3OH}=0, 0.008, 0.016, 0.024) in water (see Table S1). Samples are arranged in orthogonal simulation boxes, elongated along the *z*-axis, and are composed of two phases. The first phase is an aqueous solution of solvated methane and methanol at the given respective concentrations, and it is in the central part of the simulation box with respect to the *z*-axis. The second is a methane liquid phase located on the sides of the periodic simulation box with respect to the *z*-axis. The interfaces between the two phases are flat and perpendicular relative to the *z*-axis. For all simulation boxes, the water-methane interface was positioned at $z_1 = -42$ Å and $z_2 = 42$ Å. Further, we denominate φ the central orthogonal sub-volume between $z_{\varphi 1} = -32$ Å and $z_{\varphi 2} = 32$ Å on which we impose a prescribed concentration of methane and methanol. All samples were filled up randomly with the methane and water molecules, with numbers specified in Table S1 respecting the arrangement of the two phases and the target values of methane and methanol concentration in φ, as mentioned previously. The overall size of the simulation box is ~52 x 52 x 110 Å.

D-NEMD simulations were run in the constant number of particles, pressure and temperature NPT ensemble, for which we used the modified form of the Melchionna *et al.* barostat.[16] All of

the simulations were performed using our in-house modified version of DL_POLY Classic package.[17] During NPT D-NEMD relaxation simulations, the positions $z_{\varphi i}$ of the two perpendicular flat boundaries delimiting the sub-cell φ were evolved in time, t, following the relation $z_{\varphi i}(t) = (z_{cell}(t) / z_{cell}(t_0)) \cdot z_{\varphi i}(t_0)$, where z_{cell} denotes the side of the simulation box along the z-axis, and t_0 is the initial time of the simulation.

Table S1: Methane composition of systems simulated; the capital letter of the label refers to the methane mole fraction, whilst the following lower-case letter denotes the methanol mole fraction.

Label	No. of Trajectories	Methane molar fraction χ_{CH4} in solution	Total No. of water molecules	Total No. of methane molecules	No. of restrained methane molecules	No. of (restrained) methanol molecules	Methanol mole fraction χ_{CH3OH} in solution
**Aa**	80	0.038	7800	1280	240	0	0
**Ab**	80	0.038	7800	1280	240	48	0.008
**Ac**	40	0.038	7800	1280	240	92	0.016
**Ad**	40	0.038	7800	1280	240	140	0.024
**Ba**	64	0.044	6847	1153	240	0	0
**Bb**	64	0.044	6847	1153	240	48	0.008
**Bc**	40	0.044	6847	1153	240	92	0.016
**Bd**	40	0.044	6847	1153	240	140	0.024
**Ca**	64	0.052	6847	1153	276	0	0
**Cb**	64	0.052	6847	1153	276	48	0.008
**Cc**	40	0.052	6847	1153	276	92	0.016
**Cd**	40	0.052	6847	1153	276	140	0.024
**Da**	40	0.058	6847	1153	312	0	0
**Db**	40	0.058	6847	1153	312	48	0.008
**Dc**	40	0.058	6847	1153	312	92	0.016
**Dd**	40	0.058	6847	1153	312	140	0.024

The Methanol Model.

In this article we introduced a simple model for methanol, which is compatible and consistent with the water/methane model proposed by Jacobson and Molinero.[18] Our methanol molecule consists of the rigid union between a mW water unit, a point particle, and a united-atom CH_4 unit. The mW unit is a Stillinger-Weber point particle with a typical tetrahedral coordination with other mW units, modeling the typical tetrahedral coordination among water molecules in liquid water, ices and clathrates. The united-atom CH_4 unit interacts with mW water molecules

through the two-body repulsive term of the Stillinger-Weber potential; the parameters of this interaction have been tuned to reproduce some of the characteristics of methane/water mixtures.[18] Thus, bonding a CH_4 unit to an mW one prevents the formation of any hydrogen bonding with other mW water units along the mW-CH_4 direction, which reduces the maximum number of hydrogen bonds that can be formed by the methanol to three, like for real CH_3OH. In addition, this simplistic methanol model preserves the characteristic to be made of a hydrophilic head and a (short) hydrophobic tail. Summarizing, our mW-CH_4 methanol model, though very simple, presents the main characteristics of the real molecule.

To validate this model, we performed MD simulations of i) water/methanol solutions and ii) sI methane clathrate hydrate incorporating methanol in the structure. These calculations have been performed with both the models used in present work and with all-atoms force fields. For all-atom calculations we used TIP3P and TIP4P water,[19] and OPLS[20,21] methane and methanol.

To validate the methanol model used in the present work we compare methanol oxygen-water oxygen (partial) pair correlation function *g(r)* in the liquid water/methanol mixture and sI methane clathrate incorporating methanol (Fig. S1). This allows us to validate the capability of our simple but effective CH_4-mW methanol model to mimic the hydrogen bonding of all-atoms force field both in terms of structure and energetics. Indeed, it can be shown that the pair correlation function is related to the so-called potential of the mean force,[24] also known as the Landau free energy of the distance between two atoms/particles of the given species, i.e., the effective pair interaction between methanol oxygen and water oxygen in the given system: $g(r)=exp[-U(r)/k_BT]$, $U(r)$ the effective water/methanol interaction. Fig. S1 shows a remarkably good correspondence of both the peaks position and intensity of the pair correlation function of water/methanol mixture and sI methane clathrate incorporating methanol between all-atom force field and the model used in this work. This is very appealing considering the simplicity of our methanol model and the computational efficiency one obtains when used in combination with mW/methane force models with respect to all-atom force fields. Present results confirm the suitability of the methanol, methane and water models used in this work to study the effect of the additive and its concentration on clathrate nucleation.

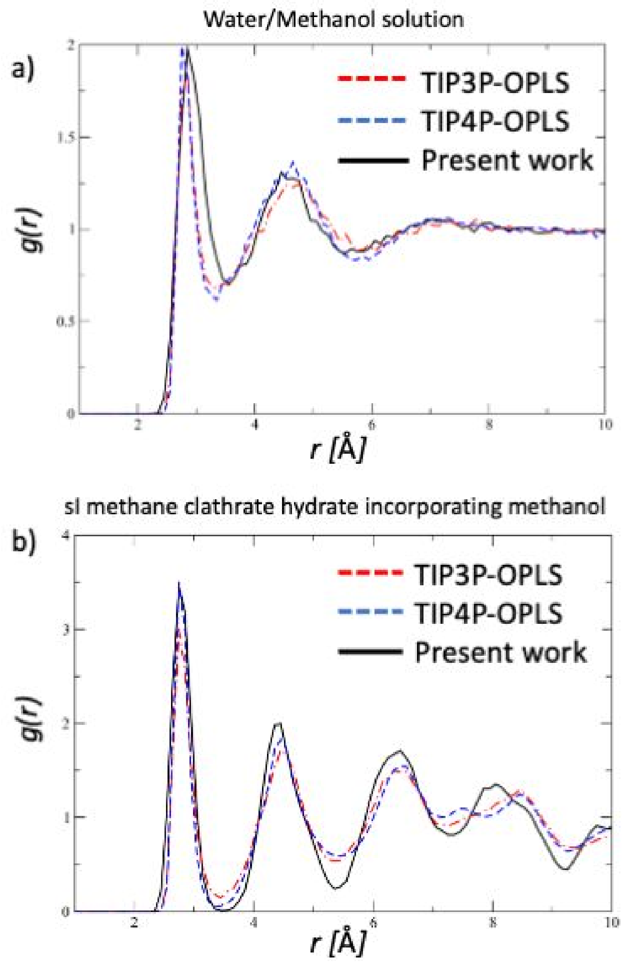

Fig S1: Methanol oxygen-water oxygen (partial) pair correlation function for a liquid water/methanol mixture (a) and sI methane clathrate incorporating methanol (b). Red and blue dashed curves represent results obtained with all-atom force fields, TIP3P or TIP4P water and OPLS methanol, while black solid line is for the water and methanol model adopted in this work.

Hydrate formation and mass-balance-based determination of hydrate-conversion yield

The experimental apparatus for hydrate formation employed a pressure vessel fabricated using 316 stainless steel with internal volume of approximately 340 cm^3 (Figs. S3 and S4). The vessel was agitated using a tilting shaker. A pressure transducer with an uncertainty of 0.2 MPa, was used to measure pressure, whilst a thermocouple with an accuracy of ±0.1 K was inserted into the cell to measure the inner temperature, with temperature/pressure readings every 2 s. Prior to each run, the vessel was cleaned thoroughly and sterilised by washing with ethanol solution (25 wt%). Each hydrate-formation experiment began with charging the equilibrium cell with approximately 20 cm^3 of deionised water. The main system was cooled to the desired temperature of 2.9 °C (to mimic seafloor temperatures), via the temperature-control system.

Once at the desired temperature, the cell was evacuated for 3 min by vacuum pump to remove any residual air, and then pressurised to the desired pressure of 120 bar using pure methane. Due to inevitable Joule-Thomson thermal contraction, the cell pressure was decreased slightly by decreasing the temperature; however, within less than 10 minutes, the temperature stabilises and remains so until the end of the chose hydrate-formation period. Then, a continuous slow pressure decline was observed during the hydrate crystal-growth stage (always under the constant-volume conditions), after nucleation. In practice, however, there is some small temperature fluctuation during hydrate formation (*i.e.*, the thermal trace of hydrate formation) due to its exothermic nature, but this is countered continually by the temperature-control system. Here, the average temperature of the production régime (*i.e.*, the temperature plateau) is considered as the starting temperature of the reaction. The system was kept at (or very near) the desired temperature of 3.5 °C for 24 hours to gauge the yield towards hydrate over this period.

The 'yield' for conversion to hydrate during formation is calculated based on the number of absorbed moles of gas into the liquid/solid phase, *i.e.*, by monitoring gas-phase pressure drop continuously on a mass-balance basis. Naturally, the first step in this number-of-gas-phase-moles-from-pressure determination lies in defining accurate the *de-facto* compressibility factor of the methane in the system, measured on our own system and tailored specifically by its slight temperature gradients and thermal inertia, in terms of the readily measurable temperature and pressure data, as detailed in the Supporting Information. Of course, we also do make use of 'reference' literature values as a function of temperature and pressure. The number of gas moles in the chamber can then be extracted using available data via $PV = znRT$.

Knowing the number of absorbed/released moles of methane, in addition to typical methane-hydrate cage-occupancy levels of 90% in the present P/T range,[25] allows for the percentage yield to methane hydrate to be calculated.

<u>Estimation of compressibility for the experimental determination of the conversion yield</u>

As mentioned above, 'yield' of conversion is calculated based on the number of absorbed moles of gas into the liquid/solid phase, which require determination of *de-facto* compressibility factor of the methane in the system. For this purpose, in five separate runs, the chamber was loaded with methane gas (and nothing else, after thorough cleaning of the type described above) at ~80 to 130 bar (spanning the entire gamut, and more, of recorded gas-phase pressure during hydrate-formation cycles) for up to five different pressure values. While all the inlet/outlet valve were closed, the reactor was heated up slowly from 3 to 5 °C whilst pressure and temperature

were recording by computer, and these data for the five points were selected to calculate the compressibility-factor value, *z*. Due to no completely and utterly definitive measurements of the number of loaded moles into the system, the working, effective *z*-value of one *P*/*T* pair was taken from ref. [26] as the correct one: the exact number of gas moles in the chamber can be extracted using this hypothesis, via $PV = znRT$. Given that this number of moles, *n*, is the same for all the other *P*/*T* points, the *de-facto z* value of those other four points can be therefore inferred via the same equation over the five independent runs, and the mean and variance taken. These sets of lowest-variance points with three coordinates (temperature, pressure and *z*-value) have been plotted as a 3D plot while the equation of a fitted surface to these points was obtained using a rational Taylor model,[27] as

$$z = \frac{z_0 + A_{01}x + B_{01}y + B_{02}y^2 + C_{02}xy}{1 + A_1x + B_1y + A_2x^2 + B_2y^2 + C_2xy}$$

- (cf. Fig. S5). This surface-fit equation was employed later to calculate the effective, operational compressibility factor of methane at various pressures and temperatures in the hydrate- formation runs from mass balances on thus-inferred gas-phase-number-of-moles data (from the gas-phase pressure) after the first 10 minutes of thermal equilibration, taking into account the temperature-variation of methane absorption in liquid with literature data for Henry's-Law constants for methane.[28]

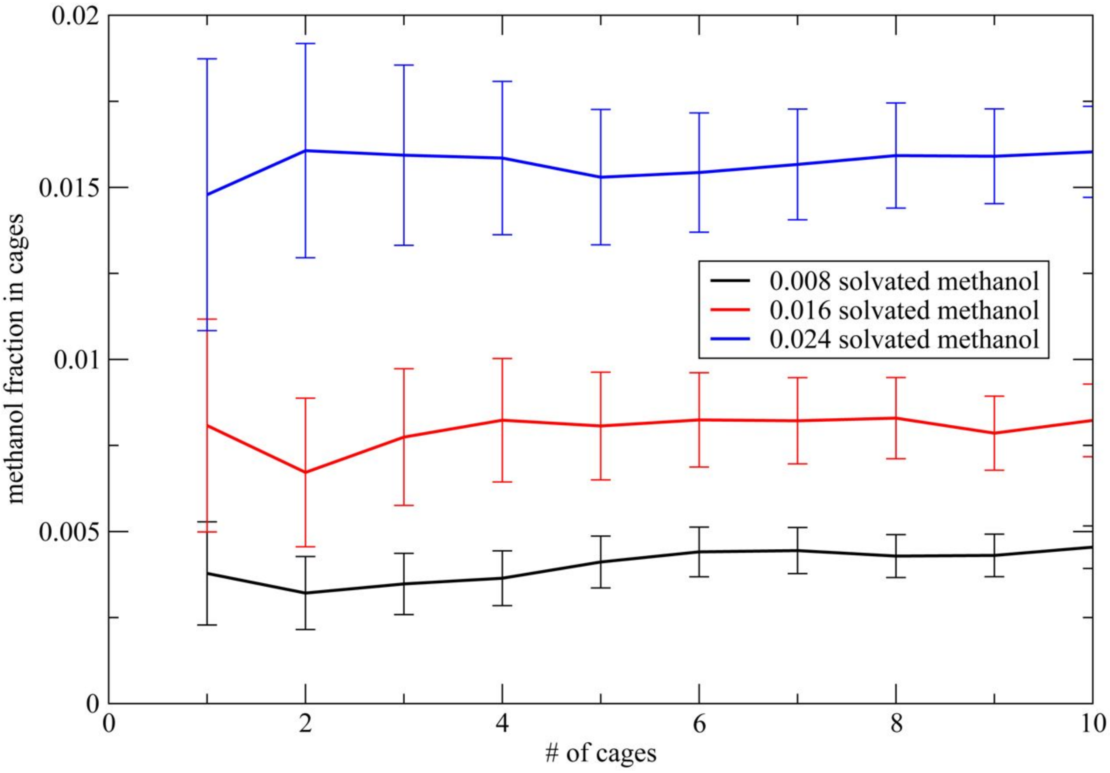

Fig S2: Methanol fraction incorporated within the clathrate hydrate nucleus as a function of the nucleus for several concentrations of methanol in solution. One notices that the fraction of methanol incorporated in the clathrate structure is almost constant along the growth of the nucleus, and this fraction grows with the concentration of methanol in solution. Since methanol forms only 3 hydrogen bonds against the 4 bonds formed by water, higher alcohol content in the clathrate nucleus implies higher hydrogen-bond defectivity, which confirm the claim of the main text.

Schematic of the hydrate-formation apparatus

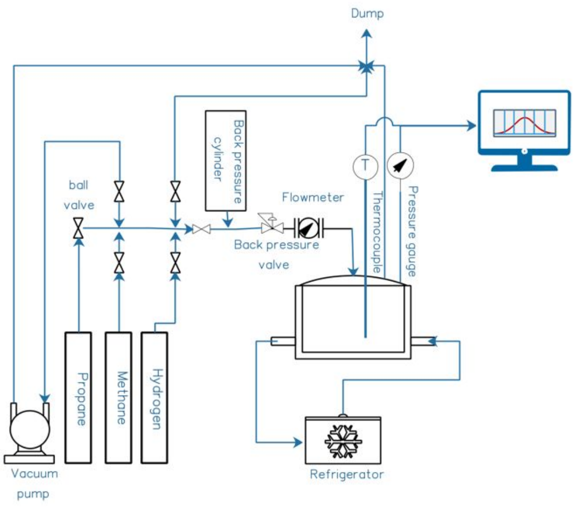

Fig S3: Schematic of gas-hydrate rig. The four main sections are: gas supplier, distribution terminal, reactor and refrigerator. High-purity (N5-level) gases (methane, propane, CO_2 and hydrogen) are supplied to the 0.3 litre, 200 bar-rated stainless-steel and rocker-mounted pressure vessel through the distribution terminal, with line-cleaning before purging the desired gas, by way of mass-flow controller and accurate measurement of gas loading into the (liquid-/sediment-loaded) reactor. The system operates under either isobaric or constant-volume modes, with a back-pressure cylinder for isobaric operation. For the constant-volume case, the reactor's inlet valve is closed upon reaching the desired pressure, and pressure logged digitally every 2-10 s for the experiment's duration (as in the current study).

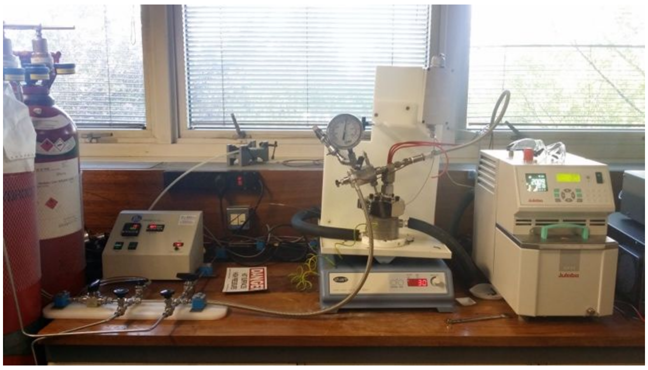

Fig. S4: Photograph of current system, showing pressure vessel mounted on the rocker, gas tanks, and the temperature-control system (on right).

<u>Surface fit for Compressibility factor</u>

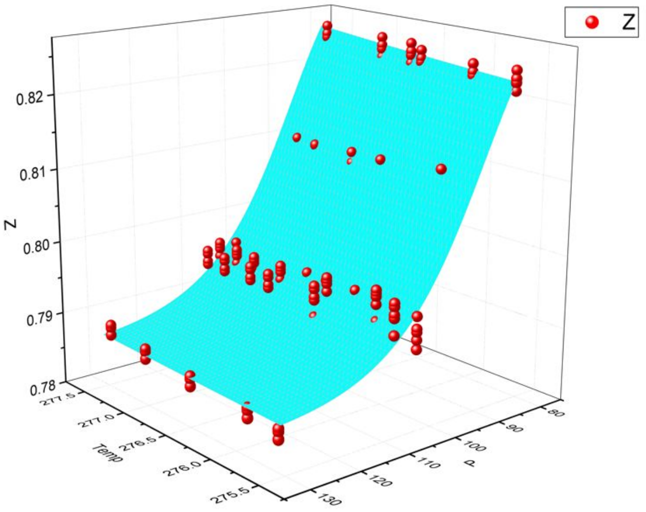

Fig. S5: Three coordinates (temperature - *x* (K), pressure - *y* (bar) and the dependent *z*-value) as a three-dimensional plot, showing the fitted surface via a rational Taylor model,[27] with quality-of-fit data below:

$$z = \frac{z_0 + A_{01}x + B_{01}y + B_{02}y^2 + C_{02}xy}{1 + A_1x + B_1y + A_2x^2 + B_2y^2 + C_2xy}$$

	Z
Number of Points	183
Degrees of Freedom	173
Reduced Chi-Sqr	4.41374E-6
Residual Sum of Squares	7.63577E-4
R-Square(COD)	0.97527
Adj. R-Square	0.97399
Fit Status	Succeeded(100)

ANOVA table:

	DF	Sum of Square	Mean Square	F Value
Regression	10	117.54216	11.75422	2.6631E6

Residual	173	7.63577E-4	4.41374E-6	
Uncorrected Tot	183	117.54292		
Corrected Tota	182	0.03088		

Parameters:

	Value	Standard Errc	t-Value	Prob>|t|	Dependency
z0	127.85149	480.58194	0.26603	0.79053	1
A01	0.18459	3.67999	0.05016	0.96005	1
B01	-1.42914	6.2184	-0.22982	0.8185	1
B02	0.02352	0.06358	0.36991	0.7119	1
C02	-0.00898	0.01872	-0.47965	0.63208	1
A1	1.2883	1.59834	0.80602	0.42134	1
A2	-0.00177	0.00751	-0.23546	0.81413	1
B1	-1.49351	6.9701	-0.21427	0.83059	1
B2	0.02963	0.08104	0.36556	0.71514	1
C2	-0.01231	0.02989	-0.41181	0.68099	1

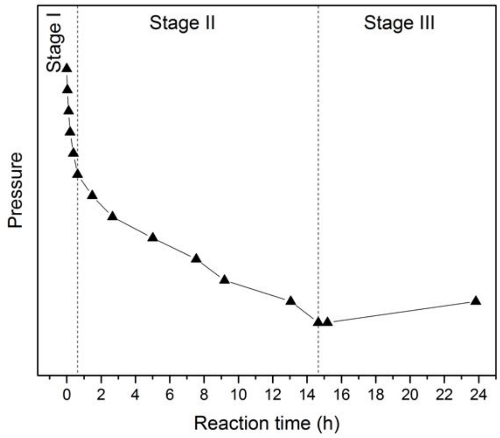

Fig. S6: Typical example of pressure evolution during 24-hour hydrate-formation processes in the presence of methanol, redolent of that of propane-hydrate formation in methanol.

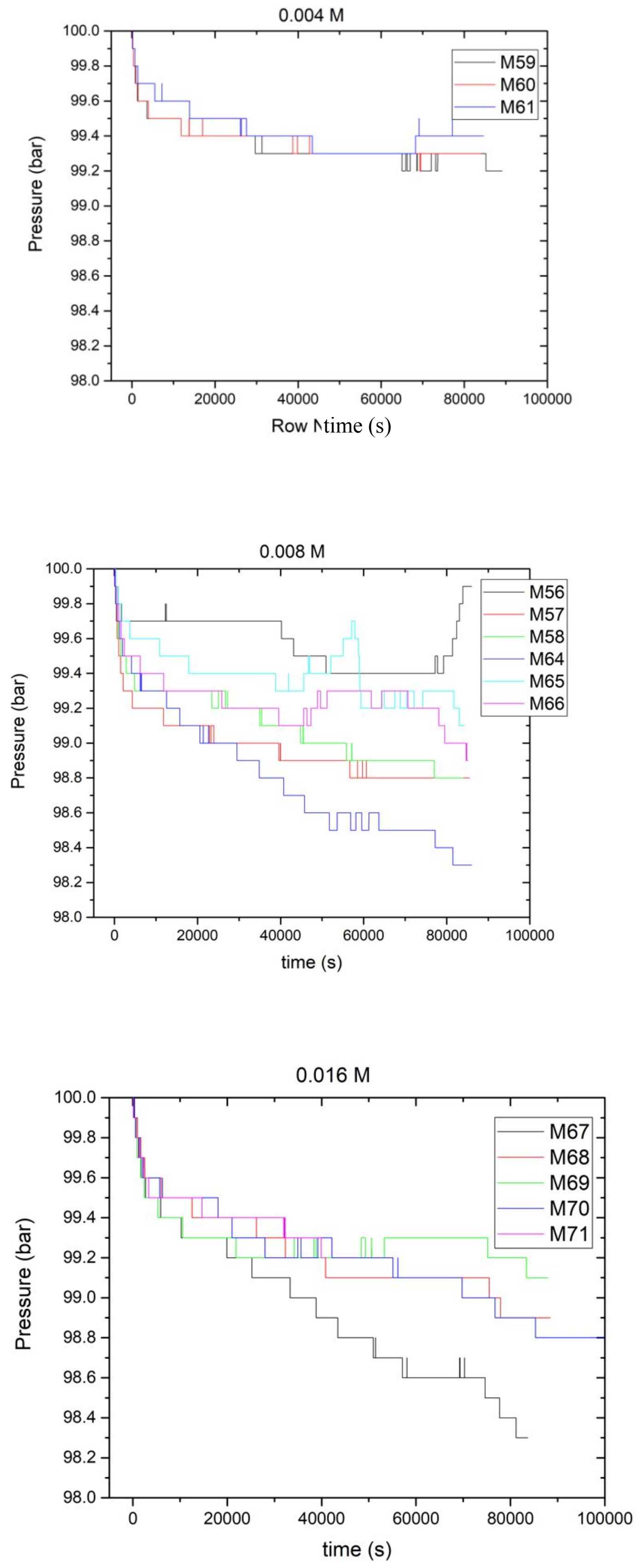

Fig. S7: Pressure-evolution statistics during 24-hour hydrate-formation processes in the presence of methanol for 0.004, 0.008, 0.016 methanol mole fractions.

Mean first passage times from restrained MD as a function of methane and methanol mole fractions

Ensemble 0.044 in molar fraction of methane without methanol

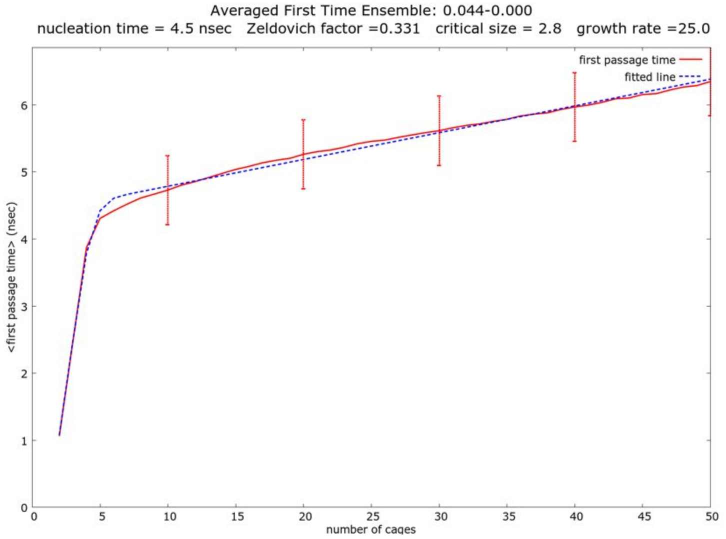

Ensemble 0.044 in molar fraction of methane with methanol at 0.008 in molar fraction

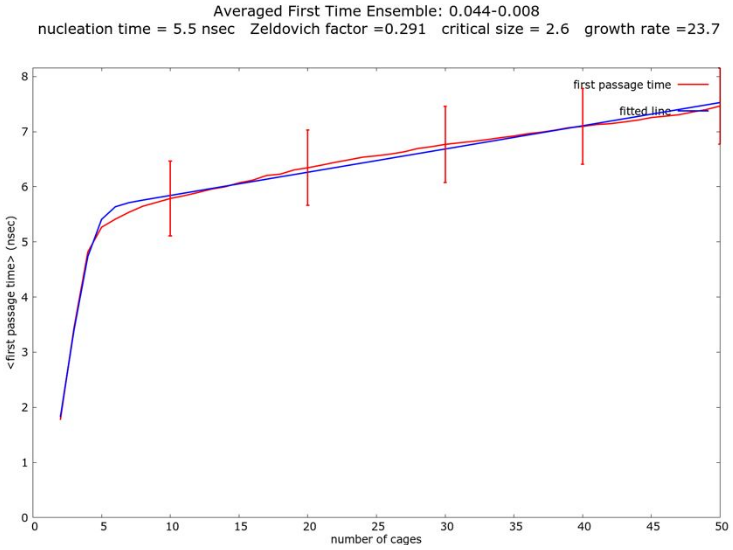

Ensemble 0.044 in molar fraction of methane with methanol at 0.016 in molar fraction

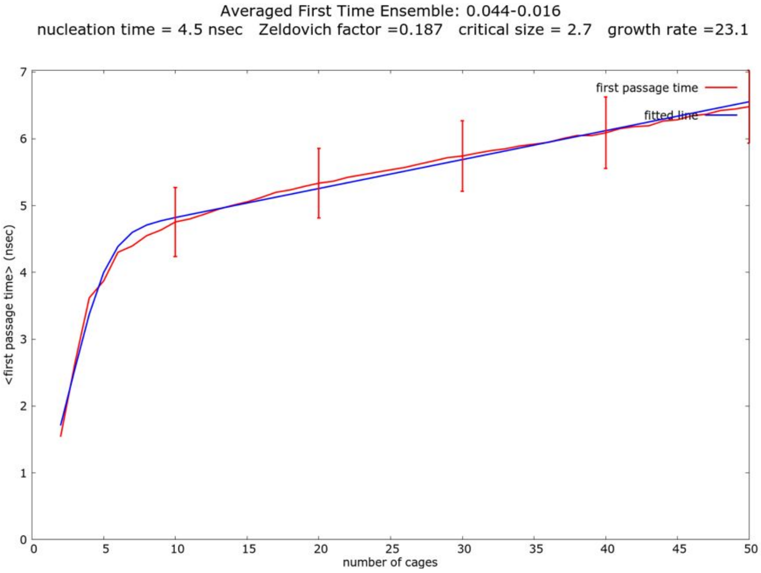

Ensemble 0.052 in molar fraction of methane without methanol

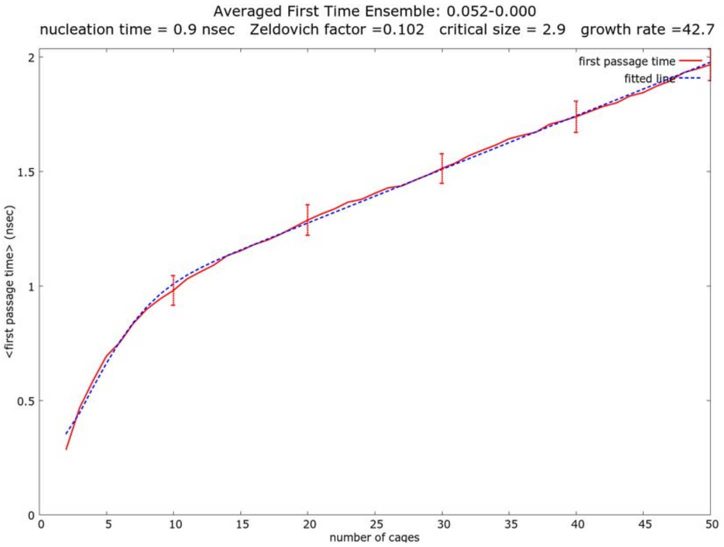

Ensemble 0.052 in molar fraction of methane with methanol at 0.008 in molar fraction

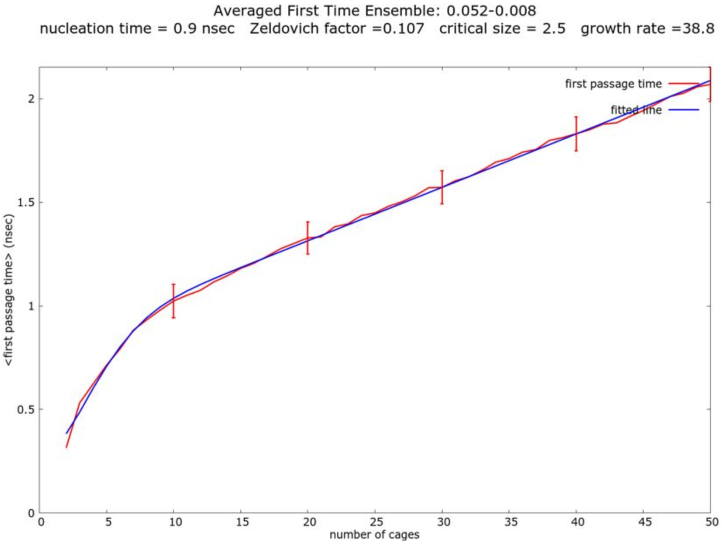

Ensemble 0.052 in molar fraction of methane with methanol at 0.016 in molar fraction

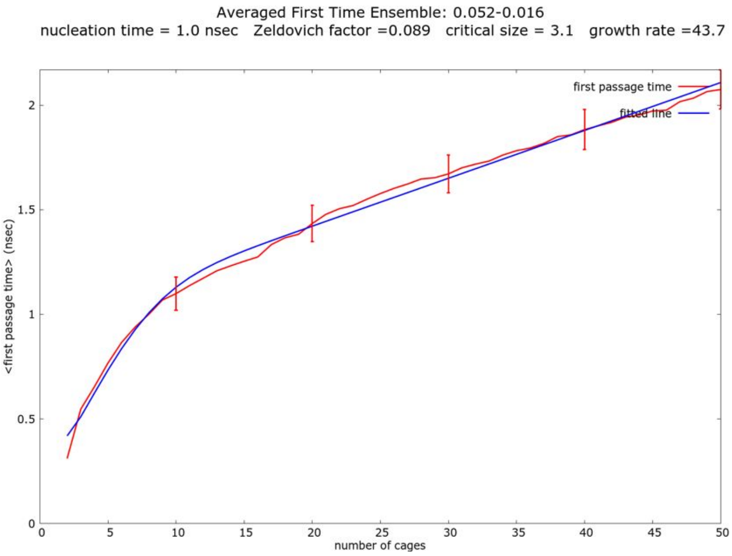

Ensemble 0.058 in molar fraction of methane without methanol

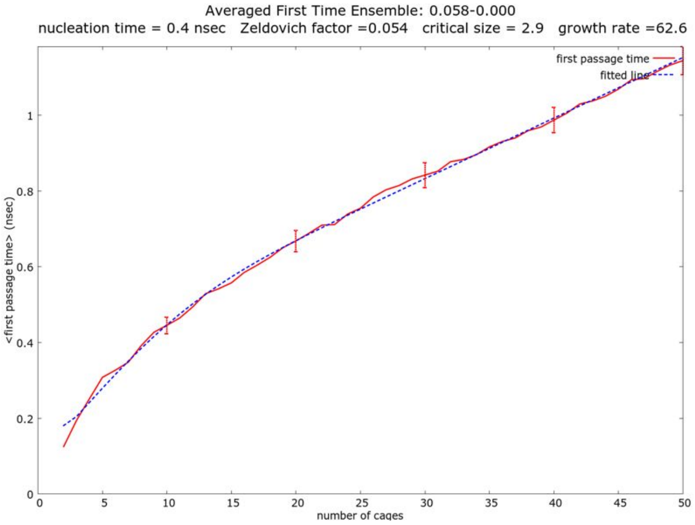

Ensemble 0.058 in molar fraction of methane with methanol at 0.008 in molar fraction

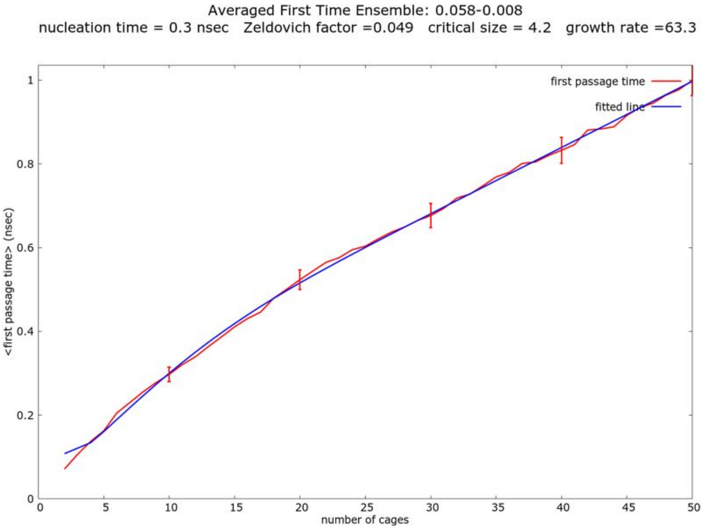

Ensemble 0.058 in molar fraction of methane with methanol at 0.016 in molar fraction

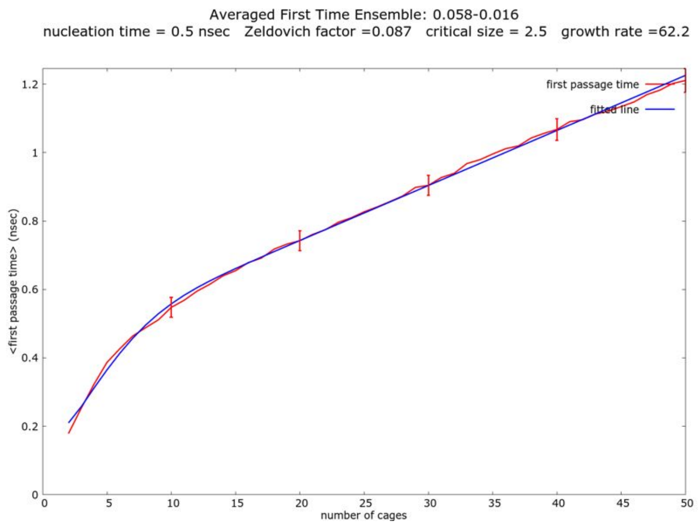

Fig. S8: Mean first passage times as a function of hydrate-nucleus size from restrained MD as a function of methane and methanol mole fractions

Table S2: Hydrate-yield data (%) for experiments at essentially the same molar methanol concentrations as those in molecular simulation; see also Figs. 2 & S6.

Methanol Mole Fraction	Yield (%)						
	Run 1	Run 2	Run 3	Run 4	Run 5	Run 6	Average Yield (%)
0.004	8	7	8	-	-	-	7.7±0.5
0.008	7	12	13	19	10	11	12.0±3.7
0.016	17	12	10	12	9	12	12.0±2.5

REFERENCES

(1) Ciccotti, G.; Jacucci, G.; McDonald, I. R. "Thought-Experiments" by Molecular Dynamics. *J. Stat. Phys.* **1979**, *21* (1), 1–22. https://doi.org/10.1007/BF01011477.

(2) Orlandini, S.; Meloni, S.; Ciccotti, G. Hydrodynamics from Statistical Mechanics: Combined Dynamical-NEMD and Conditional Sampling to Relax an Interface between Two Immiscible Liquids. *Phys. Chem. Chem. Phys.* **2011**, *13* (29), 13177. https://doi.org/10.1039/c1cp20778d.

(3) Maragliano, L.; Vanden-Eijnden, E. A Temperature Accelerated Method for Sampling Free Energy and Determining Reaction Pathways in Rare Events Simulations. *Chem. Phys. Lett.* **2006**, *426* (1–3), 168–175.

https://doi.org/10.1016/j.cplett.2006.05.062.
(4) Bonella, S.; Meloni, S.; Ciccotti, G. Theory and Methods for Rare Events. *Eur. Phys. J. B* **2012**, *85* (3), 97. https://doi.org/10.1140/epjb/e2012-20366-2.
(5) Ciccotti, G.; Meloni, S. Temperature Accelerated Monte Carlo (TAMC): A Method for Sampling the Free Energy Surface of Non-Analytical Collective Variables. *Phys. Chem. Chem. Phys.* **2011**, *13* (13). https://doi.org/10.1039/c0cp01335h.
(6) Meloni, S.; Ciccotti, G. Free Energies for Rare Events: Temperature Accelerated MD and MC. *Eur. Phys. J. Spec. Top.* **2015**, *224* (12). https://doi.org/10.1140/epjst/e2015-02418-7.
(7) Irving, J. H.; Kirkwood, J. G. The Statistical Mechanical Theory of Transport Processes. IV. The Equations of Hydrodynamics. *J. Chem. Phys.* **1950**, *18* (6), 817–829. https://doi.org/10.1063/1.1747782.
(8) Orlandini, S.; Meloni, S.; Colombo, L. Order-Disorder Phase Change in Embedded Si Nanoparticles. *Phys. Rev. B - Condens. Matter Mater. Phys.* **2011**, *83* (23). https://doi.org/10.1103/PhysRevB.83.235303.
(9) Orlandini, S.; Meloni, S.; Ciccotti, G. Combining Rare Events Techniques: Phase Change in Si Nanoparticles. *J. Stat. Phys.* **2011**, *145* (4). https://doi.org/10.1007/s10955-011-0390-9.
(10) Lauricella, M.; Meloni, S.; English, N. J.; Peters, B.; Ciccotti, G. Methane Clathrate Hydrate Nucleation Mechanism by Advanced Molecular Simulations. *J. Phys. Chem. C* **2014**, *118* (40). https://doi.org/10.1021/jp5052479.
(11) Lauricella, M.; Ciccotti, G.; English, N. J.; Peters, B.; Meloni, S. Mechanisms and Nucleation Rate of Methane Hydrate by Dynamical Nonequilibrium Molecular Dynamics. *J. Phys. Chem. C* **2017**, *121* (39). https://doi.org/10.1021/acs.jpcc.7b05754.
(12) Lauricella, M.; Meloni, S.; Liang, S.; English, N. J.; Kusalik, P. G.; Ciccotti, G. Clathrate Structure-Type Recognition: Application to Hydrate Nucleation and Crystallisation. *J. Chem. Phys.* **2015**, *142* (24). https://doi.org/10.1063/1.4922696.
(13) Ripmeester, J. A.; Alavi, S. Molecular Simulations of Methane Hydrate Nucleation. *ChemPhysChem* **2010**, *11* (5), 978–980. https://doi.org/10.1002/cphc.201000024.
(14) RODGER, P. M. Methane Hydrate: Melting and Memory. *Ann. N. Y. Acad.*

Sci. **2006**, *912* (1), 474–482. https://doi.org/10.1111/j.1749-6632.2000.tb06802.x.

(15) Mezei, M.; Speedy, R. J. Simulation Studies of the Dihedral Angle in Water. *J. Phys. Chem.* **1984**, *88* (15), 3180–3182. https://doi.org/10.1021/j150659a008.

(16) Melchionna, S.; Ciccotti, G.; Lee Holian, B. Hoover NPT Dynamics for Systems Varying in Shape and Size. *Mol. Phys.* **1993**, *78* (3), 533–544. https://doi.org/10.1080/00268979300100371.

(17) Smith, W.; Forester, T. R. DL-POLY-2.0: A General-Purpose Parallel Molecular Dynamics Simulation Package. *J. Mol. Graph.* **1996**, *14* (3), 136–141. https://doi.org/10.1016/S0263-7855(96)00043-4.

(18) Jacobson, L. C.; Molinero, V. A Methane−Water Model for Coarse-Grained Simulations of Solutions and Clathrate Hydrates. *J. Phys. Chem. B* **2010**, *114* (21), 7302–7311. https://doi.org/10.1021/jp1013576.

(19) Jorgensen, W. L.; Chandrasekhar, J.; Madura, J. D.; Impey, R. W.; Klein, M. L. Comparison of Simple Potential Functions for Simulating Liquid Water. *J. Chem. Phys.* **1983**, *79* (2), 926–935. https://doi.org/10.1063/1.445869.

(20) Jorgensen, W. L.; Tirado-Rives, J. The OPLS [Optimized Potentials for Liquid Simulations] Potential Functions for Proteins, Energy Minimizations for Crystals of Cyclic Peptides and Crambin. *J. Am. Chem. Soc.* **1988**, *110* (6), 1657–1666. https://doi.org/10.1021/ja00214a001.

(21) Jorgensen, W. L.; Maxwell, D. S.; Tirado-Rives, J. Development and Testing of the OPLS All-Atom Force Field on Conformational Energetics and Properties of Organic Liquids. *J. Am. Chem. Soc.* **1996**, *118* (45), 11225–11236. https://doi.org/10.1021/ja9621760.

(22) Perdew, J. P.; Burke, K.; Ernzerhof, M. Generalized Gradient Approximation Made Simple. *Phys. Rev. Lett.* **1996**, *77* (18), 3865–3868. https://doi.org/10.1103/PhysRevLett.77.3865.

(23) Löwdin, P. On the Non-Orthogonality Problem Connected with the Use of Atomic Wave Functions in the Theory of Molecules and Crystals. *J. Chem. Phys.* **1950**, *18* (3), 365–375. https://doi.org/10.1063/1.1747632.

(24) Chandler, D. *Introduction to Modern Statistical Mechanics*; Oxford University Press: New York, Oxford.

(25) Makogon, Y. F. *Hydrates of Hydrocarbons*; PennWell Books: Tulsa, Oklahoma, 1997.

(26) B. Wischnewski. Peace Software.

(27) OriginLab. Origin Graphing/Analysis Software. Northampton, Massachusetts.

(28) Sander, R.; Acree, W. E. J.; Chickos, J. S. Henry's Law Constants. In *NIST Chemistry WebBook, NIST Standard Reference Database Number 69*; Linstrom, P. J., Mallard, W. G., Eds.; National Institute of Standards and Technology: Gaithersburg MD, 20899, USA.
